# Crystal Structure of the Catalytic and Cytochrome *b* Domains in a Eukaryotic Pyrroloquinoline Quinone-Dependent Dehydrogenase

**DOI:** 10.1128/AEM.01692-19

**Published:** 2019-11-27

**Authors:** Kouta Takeda, Takuya Ishida, Makoto Yoshida, Masahiro Samejima, Hiroyuki Ohno, Kiyohiko Igarashi, Nobuhumi Nakamura

**Affiliations:** a Department of Biotechnology and Life Science, Tokyo University of Agriculture and Technology, Tokyo, Japan; b JEM Utilization Center, Japan Aerospace Exploration Agency, Ibaraki, Japan; c Department of Environmental and Natural Resource Science, Tokyo University of Agriculture and Technology, Tokyo, Japan; d Department of Biomaterial Sciences, Graduate School of Agricultural and Life Sciences, The University of Tokyo, Tokyo, Japan; e VTT Technical Research Centre of Finland, Espoo, Finland; University of Illinois at Urbana—Champaign

**Keywords:** AA12, AA8, Carbohydrate-Active Enzymes database, *Coprinopsis cinerea*, pyrroloquinoline quinone, cytochrome *b*

## Abstract

Pyrroloquinoline quinone (PQQ) was discovered as a redox cofactor of prokaryotic glucose dehydrogenases in the 1960s, and subsequent studies have demonstrated its importance not only in bacterial systems but also in higher organisms. We have previously reported a novel eukaryotic quinohemoprotein that exhibited PQQ-dependent catalytic activity in a eukaryote. The enzyme, pyranose dehydrogenase (PDH), from the filamentous fungus Coprinopsis cinerea (*Cc*PDH) of the Basidiomycete division, is composed of a catalytic PQQ-dependent domain classified as a member of the novel auxiliary activity family 12 (AA12), an AA8 cytochrome *b* domain, and a family 1 carbohydrate-binding module (CBM1), as defined by the Carbohydrate-Active Enzymes (CAZy) database. Here, we present the crystal structures of the AA12 domain in its apo- and holo-forms and the AA8 domain of this enzyme. The crystal structures of the holo-AA12 domain bound to PQQ provide direct evidence that eukaryotes have PQQ-dependent enzymes. The AA12 domain exhibits a six-blade β-propeller fold that is also present in other known PQQ-dependent glucose dehydrogenases in bacteria. A loop structure around the active site and a calcium ion binding site are unique among the known structures of bacterial quinoproteins. The AA8 cytochrome domain has a positively charged area on its molecular surface, which is partly due to the propionate group of the heme interacting with Arg181; this feature differs from the characteristics of cytochrome *b* in the AA8 domain of the fungal cellobiose dehydrogenase and suggests that this difference may affect the pH dependence of electron transfer.

**IMPORTANCE** Pyrroloquinoline quinone (PQQ) is known as the “third coenzyme” following nicotinamide and flavin. PQQ-dependent enzymes have previously been found only in prokaryotes, and the existence of a eukaryotic PQQ-dependent enzyme was in doubt. In 2014, we found an enzyme in mushrooms that catalyzes the oxidation of various sugars in a PQQ-dependent manner and that was a PQQ-dependent enzyme found in eukaryotes. This paper presents the X-ray crystal structures of this eukaryotic PQQ-dependent quinohemoprotein, which show the active site, and identifies the amino acid residues involved in the binding of the cofactor PQQ. The presented X-ray structures reveal that the AA12 domain is in a binary complex with the coenzyme, clearly proving that PQQ-dependent enzymes exist in eukaryotes as well as prokaryotes. Because no biosynthetic system for PQQ has been reported in eukaryotes, future research on the symbiotic systems is expected.

## Abstract (precis)

Pyrroloquinoline quinone (PQQ) is known as the “third coenzyme” following nicotinamide and flavin. PQQ-dependent enzymes have previously been found only in prokaryotes, and the existence of a eukaryotic PQQ-dependent enzyme was in doubt. In 2014, we found an enzyme in mushrooms that catalyzes the oxidation of various sugars in a PQQ-dependent manner and that was a PQQ-dependent enzyme found in eukaryotes. This paper presents the X-ray crystal structures of this eukaryotic PQQ-dependent quinohemoprotein, which show the active site, and identifies the amino acid residues involved in the binding of the cofactor PQQ. The presented X-ray structures reveal that the AA12 domain is in a binary complex with the coenzyme, clearly proving that PQQ-dependent enzymes exist in eukaryotes as well as prokaryotes. Because no biosynthetic system for PQQ has been reported in eukaryotes, future research on the symbiotic systems is expected.

## INTRODUCTION

**FIG 1:**
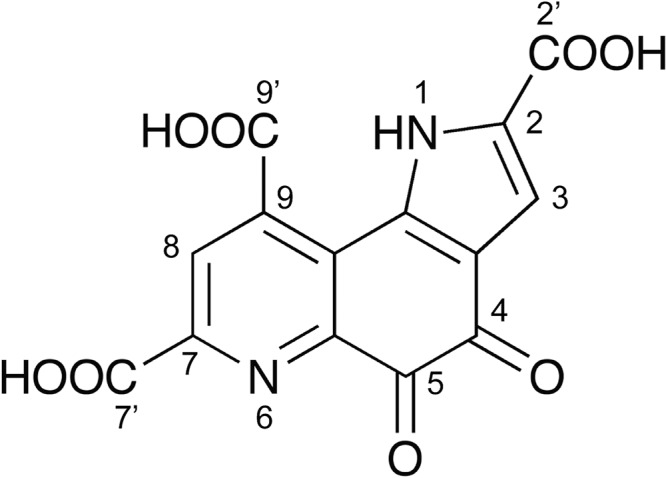
Chemical structure of PQQ.

Pyrroloquinoline quinone (PQQ) was discovered in 1964 (1). Its chemical structure (Fig. 1) was subsequently identified from experiments with bacterial methanol dehydrogenase in 1979 (2, 3). PQQ-dependent enzymes catalyze the oxidation of various sugars or alcohols in the periplasmic space in Gram-negative bacteria (4, 5). Few bacterial species are capable of synthesizing PQQ in a process that is independent of quinoprotein biosynthesis (6, 7), whereas non-PQQ-synthesizing bacteria, such as Escherichia coli, rely on the environment to supply the needed PQQ (8). There have been several reports of fungal and mammalian quinoproteins that are distinct from bacterial quinoproteins; these quinoproteins depend on other cofactors, such as topaquinone (TPQ). Akagawa et al. characterized NAD-dependent lactate dehydrogenase, which is a mammalian PQQ-binding protein in mouse NIH 3T3 fibroblasts (9). Although PQQ has nutritional importance and exerts pharmacological effects on bacteria and higher organisms (10, 11), there has not been clear evidence for a eukaryotic enzyme that utilizes PQQ as a cofactor.

**FIG 2:**
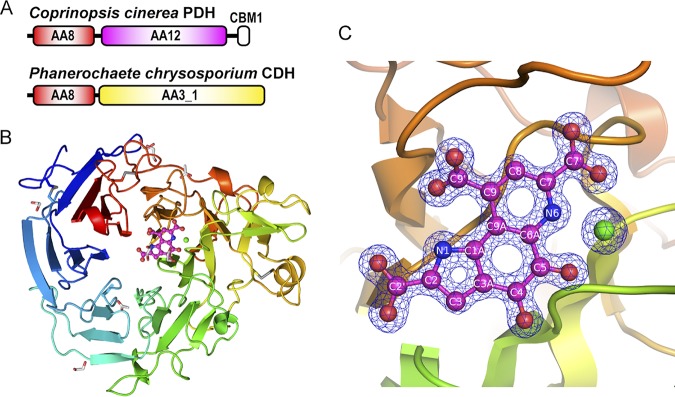
Overall structure of the AA12 domain of *Cc*PDH. (A) Domain organization of *Cc*PDH and CDH from Phanerochaete chrysosporium. Abbreviations: AA3_1, auxiliary activity (AA) family 3 subfamily 1 domain (a flavoprotein containing flavin adenine dinucleotide [FAD]); AA8, AA family 8 domain containing a *b*-type cytochrome; AA12, AA family 12 domain (a PQQ-dependent dehydrogenase domain); CBM1, family 1 carbohydrate-binding module. (B) Overall structure of the holo-AA12 domain of *Cc*PDH. The bound calcium ion is shown as a green sphere. The disulfide bonds (Cys244-Cys302, Cys492-Cys525, and Cys611-Cys619), acetate ion, and ethylene glycol molecule are shown as stick models. (C) Representation highlighting the active site at the PQQ (magenta) and the calcium ion (green) in the holo-AA12 domain, with the 2*F*_o_ − *F*_c_ electron density (where *F*_o_ and *F*_c_ are the observed and the calculated structure factors, respectively) calculated to be 1.3 Å. The atom nomenclature is indicated.

We previously found a eukaryotic PQQ-dependent pyranose dehydrogenase (PDH) from the filamentous fungus Coprinopsis cinerea (*Cc*PDH; designated *Cc*SDH in our previous paper [13]), which represented the discovery of a PQQ-dependent enzyme from a eukaryotic organism (12–14). Despite the low sequence identity (15% to 20%) to known bacterial PQQ quinoproteins, the catalytic domain of *Cc*PDH shows PQQ-dependent enzymatic activity and a high binding affinity for PQQ, having a dissociation constant (*K_d_*) of 1.1 nM. Phylogenetic analyses of the amino acid sequence of *Cc*PDH revealed the existence of a new category of PQQ quinoproteins that are present not only in fungi but also in bacteria, archaea, and amoebozoa; these proteins are significantly different from known quinoproteins. In a previous study, a protein homologous to *Cc*PDH exhibited 2-keto-d-glucose dehydrogenase (2KGDH) activity, which is a novel catalytic activity for a PQQ-dependent enzyme (15). Our findings reveal the diversity of PQQ-binding motifs and the possibility that members of the previously unknown PQQ quinoprotein family are ubiquitously distributed in all organisms. Since the enzyme has both PQQ and heme *b* (protoheme IX) prosthetic groups, it can be referred to as a quinohemoprotein. Some bacterial PQQ-dependent enzymes contain one or several *c*-type hemes and, hence, are also called quinohemoproteins (16). As shown in Fig. 2A, *Cc*PDH consists of three domains: an N-terminal cytochrome domain (auxiliary activity family 8 [AA8]), a PQQ-dependent dehydrogenase domain (AA12) in the middle of the sequence, and a C-terminal cellulose-binding domain classified as a member of the family 1 carbohydrate-binding module (CBM1). CBM1 domains are often found as molecules attached to fungal cellulolytic enzymes and are known to have the ability to adhere to crystalline cellulose in plant cell walls (17).

Known prokaryotic PQQ-dependent enzymes have a propeller superbarrel fold in common. The PQQ molecule is bound in the middle of the catalytic domain together with a catalytically essential calcium or other divalent metal ion. The cofactor is bound mainly by hydrogen bonds through its carboxyl groups. There are two types of propeller superbarrel folds: an eight-blade β-propeller structure, with each blade consisting of a four-strand antiparallel β-sheet, and a six-blade β-propeller structure (18, 19). These two types of enzymes share no amino acid sequence homology. Most of the PQQ quinoproteins adopt the former structure, an eight-blade β-propeller structure, including the soluble PQQ-dependent alcohol dehydrogenases and membrane-bound glucose dehydrogenases, whereas the latter structure has been found in a limited number of PQQ quinoproteins, such as soluble glucose dehydrogenase (sGDH) (19) and soluble aldose sugar dehydrogenase (Asd) (20, 21). As discussed in our previous report (12), the three-dimensional structure of the AA12 domain in *Cc*PDH was modeled using the Phyre2 protein fold recognition server, and the predicted structure was compared with that of other quinoproteins and structurally similar proteins. Because the AA12 domain showed low homology to the six-blade quinoproteins but no similarity to the eight-blade quinoproteins, the AA12 domain was predicted to have a six-blade, instead of an eight-blade, quinoprotein structure.

According to the Carbohydrate-Active Enzymes (CAZy) database (www.cazy.org), the cytochrome domain of *Cc*PDH is classified in subfamily 1 of auxiliary activity family 8 (AA8). AA8 domains feature an unusual fold that contains the highest β-structure content among known cytochrome structures. AA8 proteins fold into an immunoglobulin-like β-sandwich with a Met/His-coordinated *b*-type heme (22). Most of the AA8 domains discovered were found in cellobiose dehydrogenase (CDH). The AA8 domain in *Cc*PDH is similar to the AA8 domain in CDH in sequence homology and properties. The catalytic domain of *Cc*PDH contains PQQ, whereas that of CDH contains the flavin adenine dinucleotide (FAD) cofactor, and CDH is a member of the AA3 subfamily 1 (AA3_1) family (Fig. 2A). CDH is an extracellular flavocytochrome secreted by some cellulolytic fungi grown on cellulose, along with numerous other glycoside hydrolases and oxidoreductases, including lytic polysaccharide monooxygenase (LPMO) (23). LPMO oxidatively cleaves polysaccharide chains and acts synergistically with hydrolases by creating new chain ends that act as starting points for the hydrolases that catalyze the degradation (24, 25). CDH has been reported to activate LPMO and catalyze the oxidation of cellobiose in the AA3 domain, which is followed by interdomain electron transfer (IET) to the AA8 domain of CDH. Electrons are then shuttled via the AA8 domain to LPMO (26). In the catalytic cycle of *Cc*PDH, electrons are transferred from the reduced PQQ in the AA12 domain to heme *b* in the AA8 domain, which acts as a built-in mediator and transfers electrons to a heterogeneous electron acceptor. Várnai et al. reported that *Cc*PDH can activate LPMOs via the AA8 domain (27). The AA8 and CBM1 domains are unique to extracellular fungal proteins. In addition, the gene encoding *Cc*PDH is predicted to have a signal peptide sequence, suggesting that it is an extracellular oxidoreductase. Therefore, *Cc*PDH is thought to be involved in the extracellular oxidative degradation of the plant cell wall. In September 2014, the new AA12 family was created in the CAZy database, based on the discovery of *Cc*PDH, which is a PQQ-dependent enzyme found in eukaryotes. However, no structural analysis was performed. In this study, we succeeded in determining the X-ray crystal structure of both the AA12 domain (the PQQ domain) and the AA8 domain (the cytochrome domain) of *Cc*PDH. The crystal structure confirms that the enzyme is PQQ dependent by revealing an active site containing a PQQ and a calcium ion.

## RESULTS

### Structure determination.

We prepared the isolated AA8 and AA12 domains of *Cc*PDH and resolved their crystal structures individually because the crystallization of the full-length *Cc*PDH was considered to be hindered by the peptide linker regions, which are interdomain connectors. The AA12 domain lacking the AA8 domain maintains the catalytic activity at the same level as the full-length enzyme (28).

Since molecular replacement using the first diffraction data set was not successful, multiple isomorphous replacement (MIR) was used for the initial phase determination of the apo-AA12 domain crystals soaked in the mother liquor containing Pt, Au, or Hg ions. The apo-AA12 domain crystal used for the high-resolution data set diffracted to a 1.5-Å resolution, and the final model was refined to *R* and *R*_free_ values of 10.7% and 13.4%, respectively. The soaking method was not successful for obtaining the holo-form structure of the PQQ, most likely due to the way in which the apo-form of the crystal was packed. Therefore, the holo-form of the AA12 domain was prepared in solution and crystallized under different conditions. The complex of the AA12 domain with the PQQ crystal diffracted to a 1.3-Å resolution, and the final model was refined to *R* and *R*_free_ values of 15.9% and 17.7%, respectively. The AA8 domain of *Cc*PDH was determined at a 1.8-Å resolution, and the final model was refined to *R* and *R*_free_ values of 15.7% and 20.4%, respectively.

### Overall structure of the AA12 domain.

The overall structure of the AA12 domain was a six-blade β-propeller fold, as predicted by the Phyre2 server (12) and shown in Fig. 2B. The structures of the apo- and holo-AA12 domains are almost identical, with the C-α root mean square deviation (RMSD) being equal to 0.77 Å (see Fig. S1 in the supplemental material). The asymmetric unit of the holo-AA12 domain crystal is composed of two AA12 domains, each of which has a PQQ and a calcium ion, 11 ethylene glycol molecules, 2 acetate ions, and 1,520 water molecules in the asymmetric unit. The asymmetric unit of the apo-AA12 domain crystal contains a single AA12 domain (residues 241 to 649 of the full-length *Cc*PDH) with 1 calcium ion, 1 formate ion, 1 sulfate ion, 2 glycerol molecules, and 778 water molecules. A polyethylene glycol molecule is partially modeled as a triethylene glycol moiety located close to Asp601 in the apo-AA12 domain. The AA12 molecule contains 3 disulfide bonds (Cys244-Cys302, Cys492-Cys525, and Cys611-Cys619). The N-glycosylation site, Asn551, was clearly glycosylated, as predicted by the NetNGlyc server (http://www.cbs.dtu.dk/services/NetNGlyc/). The PQQ molecule was bound by the AA12 domain at the bottom of the active site, which is a small cavity near the pseudosymmetr*y* axis of the propeller fold (Fig. 2C).

### PQQ-binding site of the AA12 domain.

**FIG 3:**
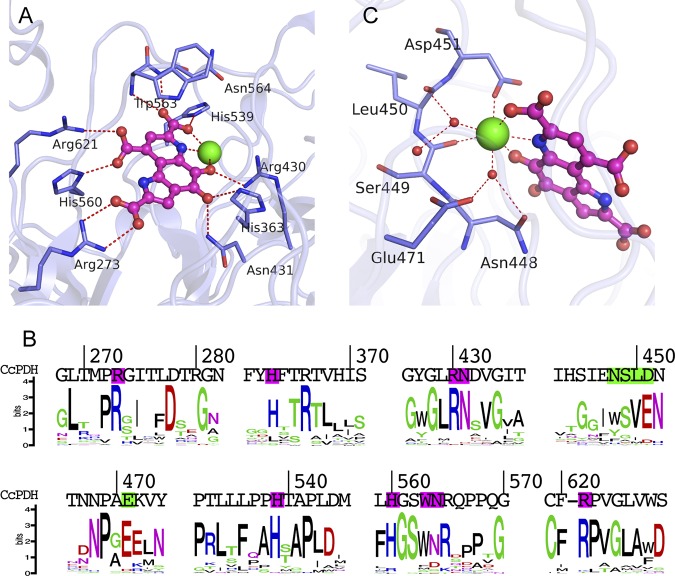
Details of the active site in the holo-AA12 domain. (A) Stick representation of the PQQ-binding site. The dashed red lines in panels A and C represent interactions within the hydrogen-bonding distance. The binding amino acid residues are named in panels A and C. (B) Illustration of the sequence homology of PQQ enzymes, which are homologous genes classified in the AA12 family. (C) Ca^2+^ binding in the active site. Water molecules are shown as red spheres.

In the known structures of bacterial PQQ quinoproteins, the PQQ molecules are tightly but noncovalently bound to the enzymes via electrostatic interactions. As shown in Fig. 3A and Fig. S2, the C-2 and C-9 carboxyl groups of the PQQ formed ion-pair interactions with Arg273 and Arg621, respectively. Furthermore, His560 was held via hydrogen bonds of the C-9 carboxyl groups of PQQ. The C-7 carboxyl groups of PQQ interact through hydrogen bonds with His539 and the amide groups of Trp563 and Asn564 on the main chain. The *ortho*-quinone O-4 was connected by a hydrogen bond to Asn431, and the side chains of His363 and the O-5 atom were bonded to Arg430 and the calcium ion. The catalytic calcium ion was also ligated to the N-6 nitrogen and the C-7 carboxyl group of the PQQ. All of these PQQ-binding residues are highly conserved among the homologous genes classified as AA12, suggesting that the PQQ bonds revealed by this structure are similar to those in other AA12 members (Fig. 3B). Hydrogen bonding by His560 is unique to the AA12 domain of *Cc*PDH and other AA12 genes.

### Calcium ion binding site of the AA12 domain.

Similar to other PQQ-dependent enzymes, *Cc*PDH requires calcium ions for its catalytic activity. In the structure presented in this study, each molecule of the AA12 domain binds one calcium ion at the active site of the PQQ molecule. As shown in Fig. 3C and Fig. S2, the calcium ion is a seven-coordinate pentagonal bipyramid with axial ligands to the side chain carboxylate of Asp451 and a water molecule. The carbonyl oxygen atoms of Ser449 in the main chain, a water molecule, and the PQQ are on the pentagonal plane. The binding residue of Ser449 is relatively well preserved on the AA12 family, while Asp451 is often less preserved and is more often glutamic residue, which has the same functional group (Fig. 3B). Although Acinetobacter calcoaceticus sGDH and E. coli Asd have the same pentagonal bipyramid calcium-binding sites for *Cc*PDH, the binding residues were not conserved in *Cc*PDH. Asn448, Leu450, and Glu471 interact with the calcium ion via coordinated water molecules. These residues are not conserved in bacterial PQQ enzymes, in which two main-chain carbonyl groups participate in calcium ion binding. The sGDH of A. calcoaceticus binds three calcium ions per monomer, and one of the ions is in the PQQ-binding pocket (19). The other two calcium ions are located in a loop between the β-strands and thus stabilize the β-propeller fold and the dimerized enzyme (29); however, these calcium ions were not found in the *Cc*PDH structure. In the crystallized apo-AA12 domain, the calcium ion-binding site is completely obscured by adjacent molecules in the crystal symmetry; i.e., Arg606 of the symmetry mate interacts with the sulfate ion, which is a calcium ion ligand, via two hydrogen bonds (Fig. S3). This crystal packing arrangement appears to prevent PQQ binding, which explains why soaking PQQ to obtain the crystallized holostructure did not succeed.

### Overall structure of the AA8 cytochrome *b* domain.

**FIG 4:**
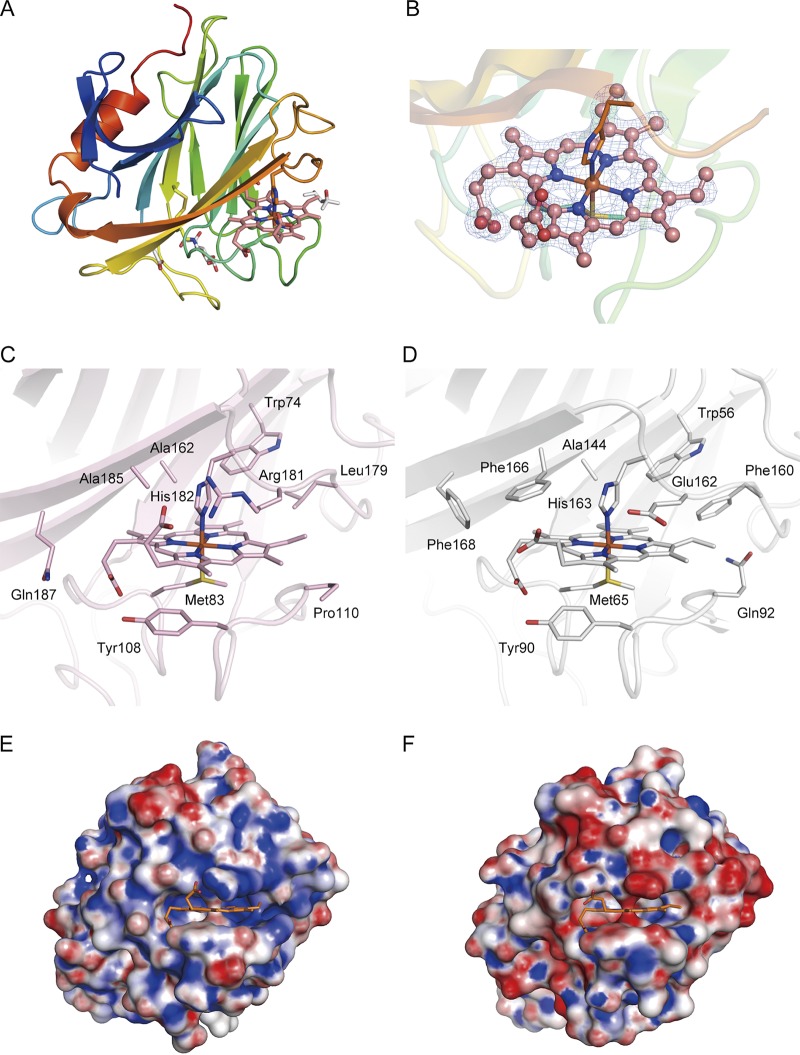
Structure of the AA8 domain of *Cc*PDH and comparison with the P. chrysosporium CDH AA8 domain. (A) Overall structure of the AA8 domain of *Cc*PDH. Here, the Met83 residue, His182 residue, disulfide bond (Cys138-Cys141), 2-methyl-2,4-pentanediol molecule, acetate ion, and GlcNAc residue are shown as stick models. (B) Close-up view of heme *b* (pink) in the AA8 domain, with the 2*F*_o_ − *F*_c_ electron density map calculated at 1.8 Å and contoured at 1.5σ. (C, D) Heme *b* binding in the AA8 domain of *Cc*PDH (C) and the *Pc*CDH AA8 domain (D) (PDB accession number 1D7D). The surrounding amino acid residues are shown as stick models with labels. (E, F) Surface charge of the AA8 domain of *Cc*PDH (E) and the *Pc*CDH AA8 domain (F). Positively charged regions are colored blue, and negatively charged regions are colored red. Molecular surfaces were drawn by use of the PyMOL APBS plug-in and color coded from red (−10 kT) to blue (+10 kT).

The AA8 domain takes the shape of an antiparallel β-sandwich fold that is very similar to that of the corresponding AA8 domain of CDHs from filamentous fungi, including that of the Phanerochaete chrysosporium CDH (*Pc*CDH; PDB accession number 1D7C) (Z-score = 27.9, RMSD = 1.3 Å, number of aligned C-α = 179) (Fig. 4A). The asymmetric unit of the AA8 domain crystal contains a single AA8 domain (residues 21 to 215 of the full-length *Cc*PDH) with a single *b*-type heme, 2 2-methyl-2,4-pentanediol molecules, 1 acetate ion, and 155 water molecules. One disulfide bond (Cys138-Cys141) that is conserved in the AA8 domain of CDHs from various filamentous fungi was observed. Asn140 is glycosylated with a single GlcNAc molecule, as predicted by the NetNGlyc (version 1.0) server.

### Heme binding pocket of the AA8 domain.

The heme iron is hexacoordinated with Met83 and His182, which serve as axial ligands, and coordination distances of approximately 2.4 Å and 2.0 Å are observed between the heme iron and the S-γ atom of Met83 and the N-σ2 atom of His182, respectively (Fig. 4B). The main-chain coordinates of the AA8 domain of *Cc*PDH closely resemble those of the AA8 domain of *Pc*CDH, whereas the amino acid residues surrounding the heme are different (Fig. 4C and D). We reported that the mutation of Phe166 to Tyr166 in *Pc*CDH significantly lowered the rate of intramolecular electron transfer from FAD to heme *b* (30). The corresponding residue in *Cc*PDH is Ala185. Other aromatic residues surrounding the heme in *Pc*CDH (Phe160 and Phe168) are absent (i.e., they are replaced by Leu179 and Gln187, respectively) in *Cc*PDH. On the other hand, Tyr108 in *Cc*PDH is conserved in CDHs and forms a hydrogen bond with the d-propionate side chain of the heme *b*. Interestingly, Glu162 in *Pc*CDH corresponds to Arg181 in *Cc*PDH, which interacts with the propionate group of the heme, which results in a local positive charge at the heme binding pocket of the AA8 domain (Fig. 4E and F).

## DISCUSSION

In this paper, we report on the structural characterization of a novel eukaryotic PQQ quinohemoprotein, *Cc*PDH. We determined the individual crystal structures of the *Cc*PDH domains: the PQQ domain (AA12) and the cytochrome domain (AA8). PQQ quinoproteins have been found to have a conserved propeller superbarrel fold structure. The crystal structure presented in this study revealed that the AA12 domain of *Cc*PDH has a 6-blade β-propeller structure. A search for the structural homology with the Dali server (http://ekhidna.biocenter.helsinki.fi/dali_server/) revealed that the AA12 domain exhibits a high degree of structural homology to bacterial PQQ quinoproteins in the Protein Data Bank (PDB), such as that of Asd in Pyrobaculum aerophilum (RMSD, 2.3 Å for C-α at position 291), despite a relatively low (<20%) sequence identity (Table 1).

**TABLE 1:** Results from the structural homology search from the Dali server^*a*^

PDB accession no.	Dali server Z-score	RMSD (Å)	Length (aa)		% identity	Enzyme	Organism
			Aligned	Total			
3A9H	32.4	2.3	291	338	19	Aldose sugar dehydrogenase	Pyrobaculum aerophilum
2ISM	32.0	2.6	290	333	18	Putative oxidoreductase (glucose dehydrogenase)	Thermus thermophilus
3DAS	31.3	2.4	288	334	16	Aldose sugar dehydrogenase	Streptomyces coelicolor
2G8S	31.0	3.0	302	347	17	Aldose sugar dehydrogenase	Escherichia coli
1CQ1	29.7	2.6	313	446	16	Glucose dehydrogenase	Acinetobacter calcoaceticus

a The structures with PDB accession numbers 3A9H (21), 2ISM (46), 2G8S (20), and 1CQ1 (19) have been described previously. aa, number of amino acids.

**FIG 5:**
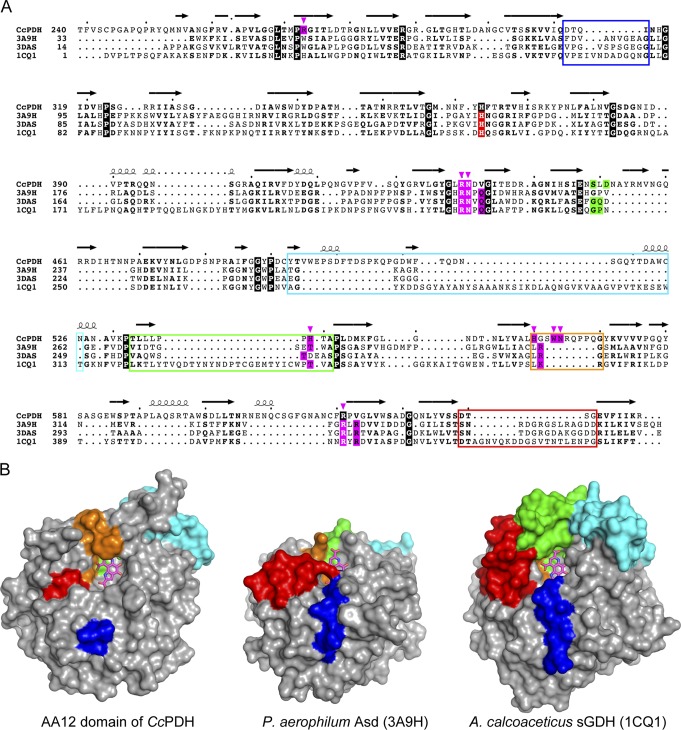
Comparison of PQQ binding in the AA12 domain and in bacterial PQQ-dependent dehydrogenases. (A) Structural alignment of the *Cc*PDH AA12 domain and bacterial PQQ-dependent dehydrogenases. Perfect matches are enclosed in boxes with a black background. Boxes with magenta and green backgrounds indicate the amino acid residues interacting with PQQ and the active-site calcium ion, respectively, via direct hydrogen bonds. The PQQ-binding residues in *Cc*PDH are indicated by arrowheads and colored magenta. Boxes with a red background indicate the proposed catalytic residues in the bacterial enzymes. Loops forming the PQQ and substrate binding sites are enclosed by colored boxes, as described in the legend to panel B. (B) Comparison of the molecular surface of the AA12 domains of *Cc*PDH (left), Asd (center; PDB accession number 3A9H), and sGDH (right). Loops forming the PQQ and substrate binding sites are colored blue, cyan, green, orange, and red, depending on the position on the β-propeller.

The structural alignment of the AA12 domain with the known bacterial PQQ-dependent dehydrogenase structures is shown in Fig. 5A. Two of the three arginines involved in PQQ binding (Arg430 and Arg621) are conserved in bacterial PQQ glucose dehydrogenases, such as sGDH and Asd. However, Arg273 is not conserved in the sequence alignment, and another Arg residue near the C terminus (e.g., Arg408 in sGDH) corresponds to an arginine residue in bacterial enzymes. The C-7 carboxyl group of PQQ forms ion-pair interactions with Lys377 of sGDH, which is absent in the AA12 domain structure. The corresponding part of the AA12 domain, instead, has a relatively longer loop that includes Trp563 and Asn564. These residues bind to the PQQ carboxyl group in the AA12 domain, which makes PQQ binding through the AA12 domain distinct from PQQ binding by known bacterial quinoproteins (Fig. 5B, surface area colored orange). Instead of loops that cover the carboxyl group of the PQQ, Asd from P. aerophilum and Asd from Streptococcus coelicolor have longer loops that form ion-pair bridges with molecules on the opposite side of the PQQ (Fig. 5B, red and blue). In addition, some of the loops (Fig. 5B, blue, cyan, green, and red) in sGDH from A. calcoaceticus are significantly longer than those in other enzymes, and this length contributes to the formation of a relatively deep active-site cavity in sGDH. The corresponding part of the *Cc*PDH AA12 domain (Fig. 5B, blue, cyan, green, red) is very short and does not contribute to the formation of the active-site cavity. PQQ is stacked on a flat and hydrophobic surface composed of Val433, Asn448, Leu543, and Ala541, which resembles the manner in which sGDH is stacked (see Fig. S4 in the supplemental material). Furthermore, the C-9 carboxyl groups of PQQ are rotated in the opposite direction from the equatorial plane, which differs from the direction of rotation for sGDH. The ethylene glycol molecules bound at the active site of the AA12 domain apparently mimic the hydroxyl groups of the bound glucose in sGDH, suggesting that *Cc*PDH shares the mechanism for binding and, consequently, the means of oxidizing pyranose with those of sGDH. The catalytic residue His144 in sGDH is conserved and corresponds to residue His363 in *Cc*PDH. However, several residues involved in substrate binding by sGDH (Gln76, Gln168, Lys169, Tyr343, and Trp346) are not conserved in *Cc*PDH (Fig. S5). The details of substrate binding by the AA12 domain of *Cc*PDH should be revealed by further analysis, e.g., using a substrate-bound structure.

Previous experiments have shown that the optimum pH for the catalytic activity of the AA12 domain (PQQ reduction) is approximately pH 6.0, whereas that for the overall activity (i.e., electron transfer through the AA8 domain) is 8.5 (28). This means that the IET from PQQ to heme *b* in *Cc*PDH is dependent on the pH and is the rate-limiting step under acidic to weakly basic conditions. For the flow of electrons to proceed in CDHs, the interdomain transfer of electrons from FAD to the heme has attracted much attention. Environmental pH affects the IET process in CDHs. We demonstrated that *Pc*CDH exhibits optimal flavin reduction of a cellobiose substrate at pH 4.5. Alternatively, the interdomain transfer of electrons from the flavin to the heme proceeds effectively at pH 3.5 and does not occur at a pH above 6.0 (31). In most basidiomycetous CDHs, IET can proceed under acidic pH conditions (32, 33). The pH dependence of the IET process has been suggested to be caused by electrostatic interactions between the associative surfaces of AA3_1 and AA8. The electrostatic repulsion between the domains at neutral and alkaline pH values would prevent the formation of the closed conformation and, consequently, lead to a decrease in the IET rate or terminate electron transport (34, 35). With a long flexible linker between both domains, CDH has two different states: one in which the AA3 domain and the AA8 domain are spatially separated (open state) and one in which the AA8 domain is docked onto the AA3 domain (closed state) (26). Using high-speed atomic force microscopy, Harada et al. directly observed an interdomain flipping motion in *Pc*CDH that involved domain-domain association and dissociation (36). IET should proceed efficiently when CDH is in the closed state, which enables suitable associations between the domains. The crystal structure analysis of the AA8 domain of *Cc*PDH revealed that Arg181 is located in the heme binding pocket and interacts with a propionate group of the heme, making the local surface positively charged around the heme, while most AA8 domains from basidiomycetous CDHs have a Glu residue at the corresponding position, and *Cc*PDH is the only family member with an Arg at that position. Because the local surface charges of the AA12 and AA8 domains of *Cc*PDH clearly differ from those of the AA3_1 and AA8 domains of *Pc*CDH, the mechanism of electrostatic interaction between the domains of *Cc*PDH is of interest for its role in IET.

In this study, we have succeeded in determining the X-ray structures of a binary complex of the AA12 domain with the cofactor to prove clearly that PQQ-dependent enzymes exist in eukaryotes as well as prokaryotes. This finding also reveals the active site and the amino acid residues involved in binding the cofactor PQQ. In conjunction with prior biochemical findings, this structural analysis effectively ends the controversy surrounding the existence of PQQ-dependent enzymes in eukaryotes. This paper presents the X-ray crystal structures of the AA8 cytochrome domain of *Cc*PDH, providing new structural insight into the electron transfer process during plant cell wall degradation by cellulolytic fungi. This research will be advanced in various fields such that the entire picture of the decomposition of woody biomass in nature will become clear.

## MATERIALS AND METHODS

### Protein preparation and crystallization.

The AA12 domain (the PQQ-dependent dehydrogenase domain, residues 240 to 649) of *Cc*PDH was heterologously expressed and purified as described previously (12). The expression construct of the AA8 domain (the cytochrome *b* domain, residues 19 to 196) of *Cc*PDH was obtained by site-directed mutagenesis using a pPICZα vector harboring the gene coding for the full-length *Cc*PDH template. PCR was carried out with KOD Plus (version 2) DNA polymerase (Toyobo, Osaka, Japan) using the following oligonucleotide pair: 5′-CCTCCGCTCTCTTGAGGTGCCCCGACC-3′ and 5′-GGTCGGGGCACCTCAAGAGAGCGGAGG-3′. The underlined residues represent the targets for substitutions that introduce a stop codon. The mutated sequence was verified by DNA sequencing with a model 3130 genetic analyzer (Applied Biosystems, Foster City, CA), and the recombinant plasmid was transformed into Pichia pastoris for subsequent protein expression according to the same protocol used for the wild-type recombinant enzyme. The recombinant AA8 domain was purified from the culture filtrate using ultrafiltration and three-step column chromatography. The culture was centrifuged (7,800 × *g* for 40 min), and the supernatant was filtered through a 100-kDa-cutoff membrane filter on a QuixStand system (GE Healthcare). After concentrating the supernatant solution with a 5-kDa-cutoff membrane filter on the QuixStand system, ammonium sulfate was added to the concentrated solution to a final concentration of 1 M, and the solution was then applied to a Toyopearl Phenyl-650S column (75-ml bed volume) equilibrated with 20 mM Tris-HCl buffer containing 1 M ammonium sulfate (pH 8.5). The proteins were eluted with a linear reverse gradient of 20 mM Tris-HCl buffer (pH 8.5) without ammonium sulfate, and the fraction exhibiting an absorbance at 430 nm was collected, desalted, and loaded on a Toyopearl DEAE-650S column (180-ml bed volume) equilibrated with 20 mM Tris-HCl (pH 8.5). The protein was eluted from the column with a linear gradient of 0 to 400 mM NaCl in the same buffer. The fraction containing the recombinant protein was collected and deglycosylated using endo-β-*N*-acetylglucosaminidase H (endo-H; New England BioLabs, Inc.). After changing the buffer to a 20 mM Tris-HCl solution (pH 8.5) using a Vivaspin 20 filter with a 5-kDa cutoff (Sartorius Japan K.K., Tokyo, Japan), the sample was loaded on a Resource Q column (6-ml bed volume) equilibrated with 20 mM Tris-HCl buffer at pH 8.5 and eluted from the column with a linear gradient of 0 to 500 mM NaCl in the same buffer. The purity of the recombinant AA8 domain was confirmed by sodium dodecyl sulfate-polyacrylamide gel electrophoresis (SDS-PAGE) and by its absorption spectrum. The protein concentration of the AA8 domain was determined by setting the absorbance to 280 nm and using an extinction coefficient of 162.1 mM^−1^ cm^−1^. The purified AA12 and AA8 domains were dissolved in 5 mM sodium acetate buffer at pH 5.0 and 5 mM Tris-HCl at pH 8.0, respectively. The apo-AA12 domain crystals were obtained with a 17.9-mg/ml protein solution by the sitting drop vapor diffusion method using a reservoir solution composed of 0.2 M sodium formate, 20% polyethylene glycol 3350, and 20 mM CaCl_2_. The sodium formate was replaced with the same concentration of potassium formate to promote crystal growth, and the crystals obtained from both conditions were used for data collection. Crystals of the AA8 domain grew in a drop composed of 4.5-mg/ml AA8 domain and 40% 2-methyl-2,4-pentanediol (MPD) that was incubated for 1 week at 20°C. The crystals of the binary complex comprising the AA12 domain with PQQ were obtained from 166-mg/ml holo-AA12 domain in solution with 50 mM sodium acetate buffer, pH 6.0, containing 1 mM CaCl_2_, which was incubated for 10 weeks at 4°C.

### Diffraction data collection, structure determination, and refinement.

X-ray diffraction data sets for the apo-AA12 and AA8 crystals were collected at beam lines BL-5A, BL17A, NE3A, and NW12A from the Photon Factory (PF) Ring and PF Advanced Ring (PF-AR) at the High Energy Accelerator Research Organization (KEK, Tsukuba, Japan). X-ray diffraction data sets for the holo-AA12 crystals were collected at beam line BL41XU at Spring-8 (Hyogo, Japan). Crystals were flash cooled with a nitrogen stream at 95 K prior to data collection. The apo-AA12 crystals were transferred to a reservoir solution containing 25% glycerol as a cryoprotectant. To obtain the heavy-atom-derivative data sets used for phase determination, the apo-AA12 crystals were soaked in reservoir solution containing 2 mM K_2_PtCl_4_, HgCl_2_, or HAuCl_4_ for 4 h. The diffraction images were processed using XDS (37). Determination of the heavy-atom sites, initial phase calculation, density modification, and initial model building were carried out using Solve/Resolve software (38), followed by automated model building by use of the PHENIX program (39). The holo-AA12 crystals were transferred to a reservoir solution containing 20% ethylene glycol as a cryoprotectant. The structure was determined by the molecular replacement method by the use of the Phaser program suite (40), using the crystal structure of the apo-form of the AA12 domain as the search model. The AA8 domain structure was determined by the molecular replacement method with the Phaser program suite, using the crystal structure of the AA8 domain of CDH from P. chrysosporium (PDB accession number 1D7D [22]) as the search model. For refinement of the models, the Coot (41), Refmac5 (42), and ARP/wARP (43) programs were used for manual rebuilding, refinement, and the addition of water molecules, respectively. The apo-AA12 domain crystals belong to space group P21 with unit cell parameters of *a *equal to 62.0 Å, *b *equal to 47.4 Å, and *c *equal to 69.1 Å. The holo-AA12 domain crystals belong to space group P212121 with unit cell parameters of *a *equal to 85.3 Å, *b *equal to 95.6 Å, and *c *equal to 106.2 Å. The X-ray diffraction data were processed with a maximum resolution of 1.3 Å. The AA8 domain crystals belong to space group P21 with unit cell parameters of *a *equal to 39.2 Å, *b *equal to 59.8 Å, and *c *equal to 40.9 Å. The X-ray diffraction data for the apo-AA12 and AA8 domains were processed with a maximum resolution of 1.5 Å and 2.0 Å, respectively. The data collection and refinement statistics are summarized in Tables 2 and 3, respectively. Searches for structural similarity were conducted, and the structural alignments were compared by use of the Dali server. Graphic images of the molecules were prepared using the PyMOL program (44), and the ESPript program (45) was used to prepare the figures.

**TABLE 2:** Data collection statistics

Date set	Values for the following^*a*^:					
	Apo-AA12	K_2_PtCl_4_	HgCl_2_	HAuCl_4_	Holo-AA12	AA8
Beamline	PF BL-5A	PF BL17A	PF-AR NE3A	PF-AR NE3A	Spring-8 BL41XU	PF-AR NW12A
Wavelength (Å)	1.00	0.98	1.00	1.00	0.9	1.00
Space group	P2_1_	P2_1_	P2_1_	P2_1_	P2_1_2_1_2_1_	P2_1_
Cell dimensions						
*a* (Å)	62.0	61.9	62.0	62.0	85.3	39.2
*b* (Å)	47.4	47.3	47.3	47.4	95.6	59.8
*c* (Å)	69.1	69.3	69.3	69.3	106.2	40.9
β (°)	115.8	116.3	116.2	116.2	90.0	90.7
Resolution (Å)	50.0–1.5 (1.59–1.50)	50.0–1.90 (1.93–1.90)	50.0–2.00 (2.03–2.00)	50.0–2.00 (2.03–2.00)	71.1–1.30 (1.38–1.30)	50.0–2.0 (2.12–2.00)
Redundancy^*c*^	5.1 (5.0)	3.6 (3.6)	3.7 (3.4)	3.7 (3.4)	3.8 (3.7)	3.3 (3.4)
No. of unique reflections	60,275	28,424	24,520	24,641	421,501	24,560
Completeness (%)	99.4 (98.3)	99.4 (99.6)	99.9 (100)	99.7 (99.8)	99.1 (95.6)	97.5 (97.5)
Average *I/*σ(*I*)^*b*^	32.9 (17.4)	12.8 (2.8)	21.1 (8.8)	21.3 (7.4)	10.8 (2.16)	8.93 (2.57)
*R*_sym_ (%)	3.6 (8.1)	11.3 (40.3)	6.9 (14.9)	6.0 (15.3)	6.2 (50.1)	7.3 (31.0)

a The values in parentheses represent those for the highest-resolution shell.

b *I*, intensity of a reflection.

c Multiplicity of observation. Number of observed reflections over number of unique reflections.

**TABLE 3:** Refinement statistics

Parameter	Value for:		
	Apo-AA12	Holo-AA12	AA8
PDB accession no.	6JT5	6JWF	6JT6
Resolution (Å)	50.0–1.50	71.07–1.30	50.0–2.0
*R*_factor_/*R*_free_ (%)	10.7/13.4	16.0/17.7	15.4/20.3
No. of reflections	56,135	202,449	12,122
RMSD from ideal values			
Bond length (Å)	0.01	0.008	0.01
Bond angle (°)	1.45	1.32	1.61
Ramachandran plot (%)			
Favored regions	95.5	95.1	94.3
Allowed regions	4.0	4.3	4.1
Disallowed regions	0.53	0.58	1.6

### Data availability.

The atomic coordinates and structure factors have been deposited in the Protein Data Bank, Research Collaboratory for Structural Bioinformatics, Rutgers University, New Brunswick, NJ (http://www.rcsb.org/), under accession numbers 6JWF, 6JT5, and 6JT6 for the holo-AA12, the apo-AA12, and the AA8 domain of *Cc*PDH, respectively.

## Supplementary Material

Supplemental file 1
