# Supplementary material for: Crystal Structure of the Catalytic and Cytochrome b Domains in a Eukaryotic Pyrroloquinoline Quinone-Dependent Dehydrogenase
Source: Appl Environ Microbiol. 2019 Nov 27;85(24):e01692-19. doi: 10.1128/AEM.01692-19 (PMC6881789; doi:10.1128/AEM.01692-19)
Supplement: Supplemental file 1 [file AEM.01692-19-s0001.pdf]

The Crystal Structure of the Catalytic Domain and the Cytochrome *b* Domain in a Eukaryotic PQQ-Dependent Dehydrogenase

Supporting Information

Kouta Takeda,[a] Takuya Ishida,[b] Makoto Yoshida,[c] Masahiro Samejima,[d] Hiroyuki Ohno,[a] Kiyohiko Igarashi,[d]# and Nobuhumi Nakamura[a]#

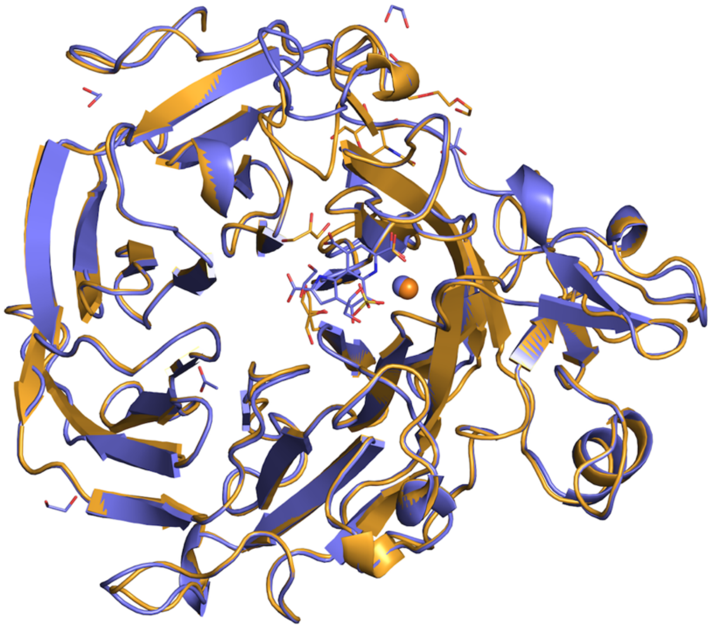

Fig. S1. Overall structure of the apo- (*bright orange*) and holo- (*blue*) AA12 domain of *Cc*PDH. The bound calcium ion is shown as a sphere. The formate ion, sulfate ion, glycerol molecule, and triethylene glycol molecule are shown as stick models in the apo-AA12 domain. PQQ, acetate ion, and ethylene glycol molecule are shown as stick models in the holo-AA12 domain.

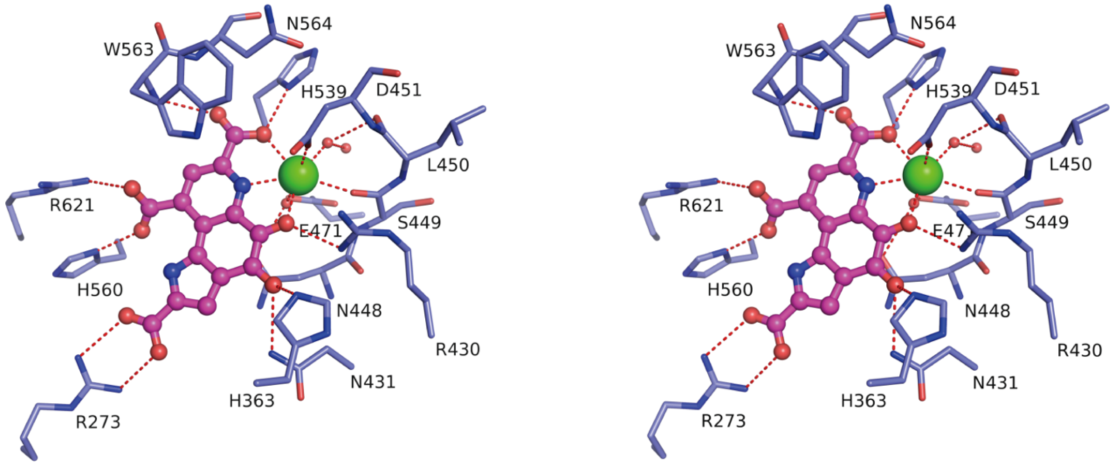

Fig. S2. Stereo view of the active site in the holo-AA12 domain of *Cc*PDH. Amino acid side-chains and water oxygens are represented by stick and small sphere, respectively. Dashed red lines indicate direct interactions within hydrogen-bonding distance.

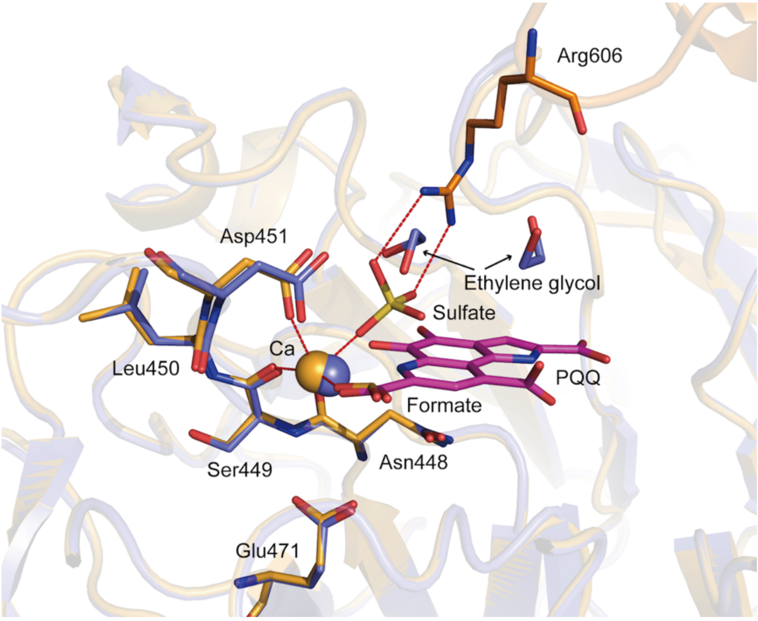

Fig. S3. Comparison of calcium ion binding by the apo- (*bright orange*) and holo- (*blue*) -AA12 domain. Calcium ion bound at the active site is shown as a sphere, and the residues involved in calcium ion binding are depicted as stick models. Arg606 of the symmetry mate (*orange*) interacts with sulfate ion. The sulfate and

formate ions are also shown as stick models and dashed red lines indicate direct interactions within hydrogen-bonding distance in the apo-AA12 domian.

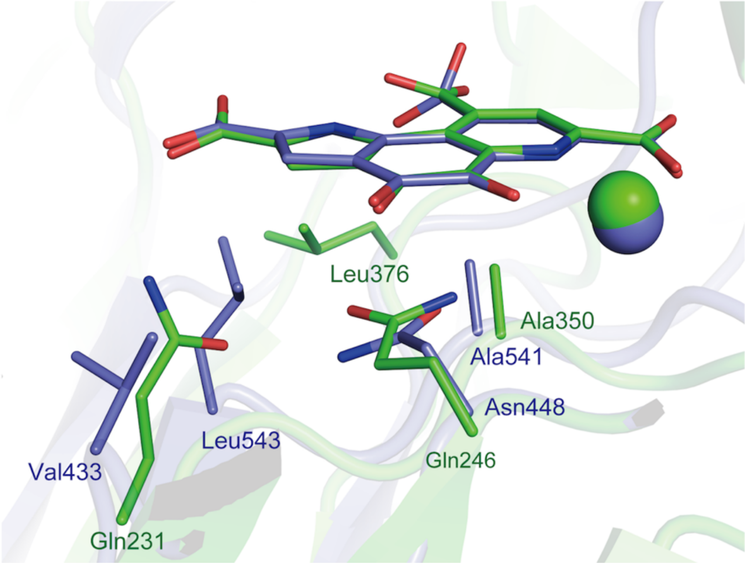

Fig. S4. The hydrophobic interaction of the bottom of PQQ of the active site in (A) the holo-AA12 domain of *Cc*PDH and (*blue*) *A. calcoaceticus* sGDH (*green*), respectively. The calcium ion is shown as a sphere, the residues are depicted as stick models. PQQ at the active site of the *Cc*PDH AA12 domain and of sGDH is shown as blue and green stick models, respectively.

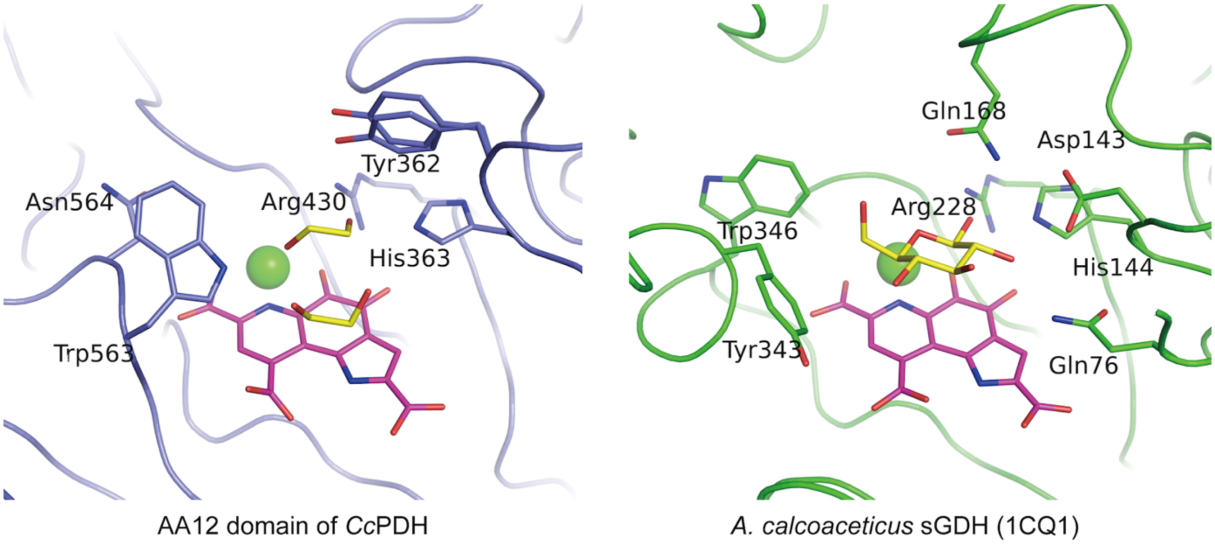

Fig. S5. The binding of ethylene glycol molecules to the active site of the AA12 domain (left) and glucose to the active site of *A. calcoaceticus* sGDH (right). The ethylene glycol and glucose molecules are shown as yellow stick models.
